# Reduced IL-17A levels in neonates born to mothers with diabetes mellitus or infection during pregnancy

**DOI:** 10.3389/fped.2025.1658039

**Published:** 2025-08-14

**Authors:** Tingting Li, Sufen Hu, Fangfang Guo, Youming He, Haiping Cheng, Mengying Wu, Yan Mao, Kexin Zhang, Juan Lin, Rui Li, Defei Ma, Shiting Li, Cheng Chen, Bing Hu, Mingbang Wang, Yingmei Xie

**Affiliations:** ^1^ Department of Neonatology, Longgang District Maternity & Child Healthcare Hospital of Shenzhen City [Affiliated Shenzhen Women and Children’s Hospital (Longgang) of Shantou University Medical College], Shenzhen, Guangdong, China; ^2^ Department of Pediatric Rehabilitation, Longgang District Maternity & Child Healthcare Hospital of Shenzhen City [Affiliated Shenzhen Women and Children’s Hospital (Longgang) of Shantou University Medical College], Shenzhen, Guangdong, China; ^3^ Maternity Ward, Longgang District Maternity & Child Healthcare Hospital of Shenzhen City [Affiliated Shenzhen Women and Children’s Hospital (Longgang) of Shantou University Medical College], Shenzhen, Guangdong, China; ^4^ Nursing Department, Longgang District Maternity & Child Healthcare Hospital of Shenzhen City [Affiliated Shenzhen Women and Children’s Hospital (Longgang) of Shantou University Medical College], Shenzhen, Guangdong, China

**Keywords:** interleukin-17A (IL-17A), umbilical cord blood, perinatal complications, gestational diabetes mellitus (GDM), perinatal infections

## Abstract

**Background/Objectives:**

Pregnancy complications are associated with adverse maternal–neonatal outcomes, but the underlying immune mechanisms remain unclear. Here we examined umbilical cord IL-17A levels and their link to gestational diabetes mellitus (GDM) and perinatal infections (PIs).

**Methods:**

This two-phase study analyzed 87 pregnant women (38 in the exploratory phase and 49 in the validation phase) from Shenzhen's Premature Infants Gut Microbiota Assembly and Neurodevelopment (PIGMAN) cohort, divided into perinatal complication (PC) (45 cases) and non-perinatal complication (NPC) (42 cases) groups. Cord blood IL-17A levels were measured by ELISA and analyzed as a continuous variable by Nonparametric rank-sum test and a categorical variable using Fisher's exact test.

**Results:**

Nonparametric analysis revealed consistently lower IL-17A levels in the PC group across both phases. The discovery phase median in the PC group was 0.67 pg/ml lower than the 5.68 pg/ml median in the NPC group (*p* = 0.001); in the validation phase, the PC and NPC group levels were 0.93 and 2.05 pg/ml (*p* = 0.012), respectively. Low IL-17A (<1 pg/ml) prevalence was significantly higher in PC cases (discovery: 61.9% vs. 11.8%, *p* = 0.002; validation: 50% vs. 36%, *p* = 0.031). Rank-sum test and Fisher's exact test demonstrated concordant results, confirming a robust association between reduced IL-17A levels and perinatal complications.

**Conclusion:**

Umbilical cord blood from PC pregnancies exhibited significantly lower IL-17A levels compared to that from NPC pregnancies, suggesting compromised neonatal cellular immunity. These findings implicate IL-17A deficiency in the immune dysregulation associated with GDM and PI. Conversely, the higher IL-17A levels observed in NPC pregnancies may reflect its protective role in maternal–fetal immunity during early development.

## 1 Introduction

Maternal exposures during pregnancy, delivery, and the early postpartum period are universally recognized perinatal risk factors that may adversely affect fetal or neonatal health. These biological, environmental, and socioeconomic factors contribute to neonatal complications, including asphyxia, infections, congenital anomalies, preterm birth, and low birth weight, with potential long-term developmental outcomes (1–4). Consequently, vigilant monitoring of perinatal risk factors carries significant clinical and public health importance. Perinatal risk factors—including recurrent pregnancy loss, recurrent implantation failure, preeclampsia, and gestational diabetes mellitus (GDM)—represent prevalent clinical challenges during the reproductive years (5–8). GDM is widely recognized as one of the most prevalent perinatal complications. The global prevalence of GDM has shown a marked increase in recent years, currently affecting 1%–14% of all pregnancies (5). GDM is significantly associated with diverse adverse fetal and maternal outcomes, including elevated risks of cesarean delivery, preeclampsia, macrosomia, and intrauterine growth restriction (6–8). Furthermore, emerging evidence links GDM to impaired neonatal neurodevelopment, which manifests as intellectual disability, language impairment, attention deficits, and behavioral disinhibition (9). Timely mitigation of perinatal risk factors may improve pregnancy success rates. However, in cases with unexplained etiology, an imbalance in immune cell profiles may be present. Indeed, maladaptive immune responses are frequently associated with pregnancy-related risk factors (10–12). These high-risk perinatal factors not only directly endanger fetal development, but may also exacerbate maternal–fetal risks by disrupting the Th1/Th2/Th17/Treg balance.

During pregnancy, the maternal–fetal interface maintains a delicate immunological equilibrium that requires precise regulation of both tolerance toward the semi-allogeneic fetus and defense against pathogens. Recent advances in our understanding of pregnancy immunology have shifted the immune regulation paradigm from the classical Th1/Th2 balance theory toward a more comprehensive Th1/Th2/Th17/Treg framework, offering novel perspectives on how perinatal risk factors influence maternal–fetal health (13). IL-17A, a pivotal cytokine produced by T helper 17 (Th17) cells, is the most well-characterized member of the Th17 cytokine family in terms of its biological functions and regulatory capacity. Emerging evidence suggests that altered IL-17A signaling may contribute to the pathogenesis of various pregnancy complications, including GDM and PI (14, 15). During pregnancy, IL-17A exhibits dual roles: it promotes inflammatory responses to combat infections while potentially disrupting immune tolerance when dysregulated. Maternal IL-17 during gestation derives mainly from CD4+ T cells in the peripheral circulation and decidual compartments (16). *In vitro* studies demonstrate that IL-17 stimulation enhances the invasive capacity of JEG-3 trophoblasts and upregulates their progesterone secretion (17). Elevated circulating IL-17 levels have been observed during the third trimester of healthy pregnancy, suggesting its potential involvement in inflammatory regulation and parturition (18). These findings imply that Th17 cells may contribute to establishing and maintaining appropriate maternal immune responses during gestation. However, the precise spatiotemporal dynamics of IL-17A expression in these clinical contexts remain poorly characterized, particularly regarding its transplacental regulation of fetal immune programming.

IL-17A plays a dual physiological role in humans. Its production appears critical for regulating bioactive molecules derived from utero-placental tissues, thereby facilitating physiological labor (19–21). Current research primarily focuses on maternal circulating IL-17A levels, while investigations into IL-17A levels during early life stages remain notably absent from the literature. IL-17A, a potential biomarker reflecting immune establishment during late gestation and early life stages, has not been sufficiently investigated in relation to perinatal complications. We hypothesized that perinatal complications of metabolic or infectious origin are associated with distinct umbilical cord blood IL-17A profiles, mirroring different immune dysregulation pathways at the maternal–fetal interface. Our findings provide novel insights into how maternal disease states may program neonatal immunity through altering the levels of this pivotal immunoregulatory cytokine.

## 2 Materials and methods

### 2.1 Study population

All study participants were recruited from The Premature Infants Gut Microbiota Assembly and Neurodevelopment (PIGMAN) Cohort Study. A total of 88 umbilical cord blood samples were collected from mother–infant pairs at Longgang District Maternal and Child Health Hospital. The study participants were categorized into two groups for blood sample collection: (1) the PC group, consisting of patients diagnosed with both GDM or PIs, and (2) the control group, comprising healthy pregnant women without any obstetric complications. GDM diagnosis was established according to standard criteria (7, 22), while perinatal infection (defined as occurring between 28 weeks of gestation and 7 days postpartum) was determined based on our study-specific criteria due to the lack of universal diagnostic standards. For the PC group, clinical infection types included histologically/clinically confirmed chorioamnionitis, intrapartum fever (≥38°C), neonatal sepsis (blood culture positivity), and congenital Toxoplasmosis, Other agents, Rubella, Cytomegalovirus, Herpes simplex virus; confirmed by serology/PCR infections (serology/PCR confirmation), with laboratory confirmation requiring at least one of the following: positive amniotic fluid culture (aerobic/anaerobic), placental pathology demonstrating neutrophilic infiltration, or positive neonatal blood/CSF culture. Clinical diagnosis of infection required maternal fever (≥38°C) plus at least two of the following: maternal leukocytosis (>15 × 10⁹/L), fetal tachycardia (>160 bpm), uterine tenderness, or purulent amniotic fluid. The control group excluded participants with maternal factors such as vaginal bleeding, menstrual disorders, allergies, smoking, or opioid use, as well as neonates with congenital/hereditary disorders or other significant abnormalities, or those who died within 24 h postpartum. All diagnostic procedures were performed by board-certified obstetricians, ensuring rigorous phenotyping of complications while minimizing confounding variables, in accordance with STROBE guidelines for observational studies, the completed STROBE checklist is provided as Supplementary Material 1. This study was approved by the Institutional Review Board of Longgang District Maternal and Child Health Hospital in Shenzhen (Ethics Approval No.: LGFYKYXMLL-2024-50).

### 2.2 Sample collection and processing

For serum preparation, whole blood was allowed to clot at 2°C–8°C overnight, followed by centrifugation at 1,000 × *g* for 20 min to collect the supernatant. For plasma isolation, EDTA-Na2 was used as an anticoagulant, with centrifugation at 1,000 × *g* for 15 min at 2 °C–8 °C within 30 min after collection to obtain the supernatant. Tissue samples were rinsed with pre-cooled PBS (0.01 M, pH 7.4), weighed and minced, then homogenized on ice in PBS containing protease inhibitors at a 1:9 (w/v) ratio, followed by freeze-thawing or ultrasonication and centrifugation at 5,000 × *g* for 5–10 min to collect the supernatant. For cell samples, adherent cells were washed with PBS, trypsinized, and centrifuged, while suspended cells were directly centrifuged. The cell pellets were washed three times with PBS and resuspended in PBS (150–200 μl per 10^6^ cells), then lysed by freeze-thawing or ultrasonication, followed by centrifugation at 1,500 × *g* for 10 min to obtain the supernatant. Body fluids and cell culture supernatants were centrifuged at 1,000 × *g* for 20 min to remove impurities. All centrifugation steps were performed at 2 °C–8 °C.

### 2.3 Serum cytokine level detection

The concentration of serum interleukin-17A (IL-17A) was determined using a human IL-17A enzyme-linked immunosorbent assay (ELISA) kit (Catalog No.: E-EL-H5812c, Elabscience Biotechnology Co., Ltd, Wuhan, China) according to the manufacturer's instructions. The calculation results showed that the intra-assay coefficient of variation (CV) for the IL-17A ELISA kit ranged from 0.09% to 1.25%, while the inter-assay CV ranged from 0.32% to 0.75%. First, microtiter plates were pre-coated with anti-human IL-17 capture antibodies, allowing the IL-17 to present in the test samples or standards to bind during incubation, while unbound components were removed through washing. Subsequently, biotinylated anti-human IL-17 detection antibodies and horseradish peroxidase–conjugated streptavidin were added sequentially, forming an immobilized immune complex through specific binding between the detection antibodies and plate-bound IL-17, as well as the high-affinity biotin-streptavidin interaction, followed by additional washing steps to eliminate unbound reagents. The enzymatic reaction was initiated by adding 3,3',5,5’-tetramethylbenzidine substrate, which produces a blue color catalyzed by HRP, and then terminated with stop solution resulting in transition to a yellow color. The optical density at 450 nm was measured using a microplate reader, and the IL-17 concentrations in the test samples were calculated from the OD450 values by extrapolating from a concurrently run standard curve. This quality-controlled procedure effectively minimized technical and instrumental variations, ensuring the inter-batch comparability of results for quantitative assessment of IL-17's pathological role in perinatal complications (Supplementary Table S1). Due to the retrospective study design requiring batch processing by clinical groups, laboratory personnel were not blinded to sample categories. However, potential bias was minimized through objective automated plate reading for IL-17A quantification, and strict adherence to standardized protocols with duplicate measurements and internal controls.

### 2.4 Statistical analysis

All statistical analyses were conducted in R (v4.2.3), primarily using the fisher.test () and wilcox.test() functions from the stats package. Normally distributed data such as gestational age and birth weight are presented as mean ± standard deviation. Non-normally distributed data such as IL-17A concentrations are presented as median (interquartile range). Given the non-normal distribution of the data, we also performed non-parametric tests [wilcox.test ()] for between-group comparisons of the original continuous data. This method does not rely on distributional assumptions and can effectively handle data with a large number of tied ranks. To address the high dispersion and significant left-skewed distribution of the IL-17A concentration data (where a substantial number of samples had measurements below the detection limit of 1 pg/ml), we dichotomized the original continuous IL-17A data: samples with IL-17A <1 pg/ml were coded as 0, and those with IL-17A ≥1 pg/ml were coded as 1. This transformation effectively resolved the statistical model applicability issues caused by the extreme skewness. Subsequently, we used Fisher's exact test [fisher.test ()] to analyze between-group differences in the binary categorical variable. This method is particularly suitable for small sample sizes and scenarios with zero-frequency cells, providing accurate probability estimates. This approach comprehensively validated the robustness of the study findings. A multiple linear regression model was employed to adjust for potential confounding factors and control for their interference with study outcomes. A significance threshold of *α* = 0.05 was set, with *p* < 0.05 considered statistically significant.

To better visualize the data distribution and intergroup differences, we employed multiple visualization methods, primarily based on ggplot2 (V3.5.2). Bar plots are used to display proportional differences in dichotomized IL-17A levels (<1 vs. ≥1 pg/ml) between groups, while boxplots illustrate the distribution of and differences in IL-17A concentrations between the PC and NPC groups. Given the non-normal distribution of the data, we overlaid individual data points on the boxplots to avoid obscuring the actual data distribution, and the statistical test results are explicitly annotated on the plots to enhance visualization.

## 3 Results

During the exploratory phase of the study, we enrolled 38 pregnant women with a mean gestational age of 39.29 ± 1.03 weeks and a mean neonatal birth weight of 3.27 ± 0.39 kg. The average IL-17A concentration was 6.12 ± 12.45 pg/ml. The exploratory cohort comprised 21 cases in the PC group (including 11 PI cases and 10 GDM cases) and 17 cases in the NPC group. In the validation phase, we recruited 49 additional participants with comparable baseline characteristics (mean gestational age: 39.23 ± 0.79 weeks; mean birth weight: 3.24 ± 0.33 kg) showing an average IL-17A level of 3.30 ± 5.80 pg/ml. The validation cohort consisted of 24 PC cases (including 7 PI cases and 17 GDM cases) and 25 NPC cases (Table 1, Supplementary Table S2).

**Table 1:** Baseline characteristics of the participants in the exploratory and validation cohorts.

Group	*N*	GA	BW	WBC (×10⁹/L)	Hb (g/L)	PLT (×10⁹/L)	LYM (%)	EOS (×10⁹/L)
DPC	21	38.71 ± 1.49	3.20 ± 0.40	10.26 ± 2.64	121.16 ± 10.97	239.47 ± 62.95	15.93 ± 5.45	0.71 ± 0.49
DNPC	17	39.47 ± 0.62	3.35 ± 0.35	9.11 ± 2.48	119 ± 10.91	221.25 ± 49.66	18.2 ± 4.33	0.74 ± 0.45
*t*/*z*		−1.96	−1.265	1.37	0.60	0.97	−1.40	−0.17
*p*		0.058	0.214	0.18	0.55	0.34	0.17	0.87
VPC	24	38.92 ± 0.97	3.28 ± 0.36	9.33 ± 2.31	118.83 ± 10.40	241.42 ± 48.91	20.13 ± 5.99	0.7 ± 0.41
VNPC	25	39.56 ± 0.82	3.28 ± 0.28	9.33 ± 2.94	106.39 ± 25.01	229.28 ± 53.04	20.62 ± 6.14	0.88 ± 0.82
*t*/*z*		−2.504	−0.008	0.004	0.097	0.832	−0.276	−0.992
*p*		0.016	0.994	0.997	0.029	0.410	0.784	0.326

GA, gestational age; BW, body weight; IL-17, interleukin-17; WBC, white blood cell (count); Hb, hemoglobin; PLT, platelet (count); LYM, lymphocyte (count); EOS, eosinophil (count); DPC, discovery period perinatal complications group; DNPC, discovery period non-perinatal complications group; VPC, validation period perinatal complications group; VNPC, validation period perinatal non-complications group.

Statistical analysis revealed that among the baseline characteristics, only gestational age showed statistically significant differences between the two groups (*p* < 0.05). To control for this potential confounding factor, we performed multiple linear regression analysis with adjustment. After adjusting for gestational age, the PC group demonstrated significantly lower IL-17 concentrations compared to the NPC group (*β* = −4.999, 95% confidence interval: −8.89 to −1.11; *p* = 0.014). However, gestational age itself showed no significant correlation with IL-17 levels (*β* = 0.026, *p* = 0.977) (Supplementary Table S3).

**Figure 1:**
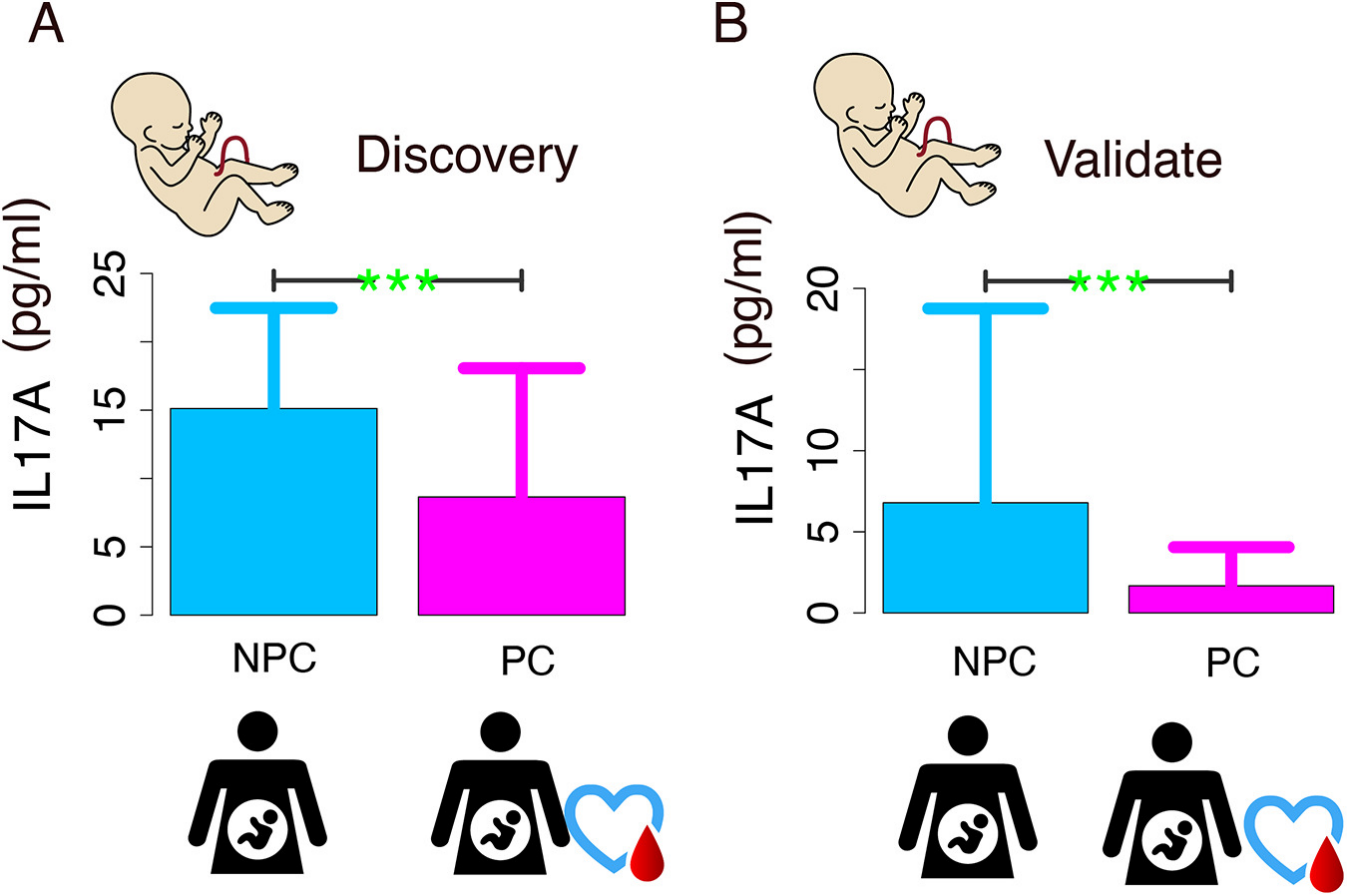
IL-17A level comparison between non-pregnant complications (NPC) and pregnant complications (PC) groups. **(A)** Discovery phase: NPC group shows significantly higher IL-17A levels (p < 0.01); **(B)** Validation phase: Elevated NPC levels are consistently observed (p < 0.01).

Both the exploratory and validation phases demonstrated characteristically right-skewed distributions, so we performed nonparametric rank-sum tests on the original continuous data. The validation phase demonstrated substantially reduced variability in IL-17A concentrations compared with the discovery phase, as evidenced by the decrease in standard deviation from 12.45 to 5.80 pg/ml, indicating greater data homogeneity. Comparative analysis of the discovery cohort demonstrated a statistically significant elevation in IL-17A concentrations in NPC subjects (median [IQR]: 5.68 [1.24, 14.7]) relative to PC controls (median [IQR]: 0.67 [0.13, 5.18]; *p* = 0.001). This statistically significant pattern was consistently reproduced in the validation cohort, with the NPC group maintaining elevated IL-17A concentrations (median [IQR]: 2.05 [0.8, 5.16]) compared with the PC group (median [IQR]: 0.93 [0.54, 1.52]; *p* = 0.021) (Figure 1).

**Figure 2:**
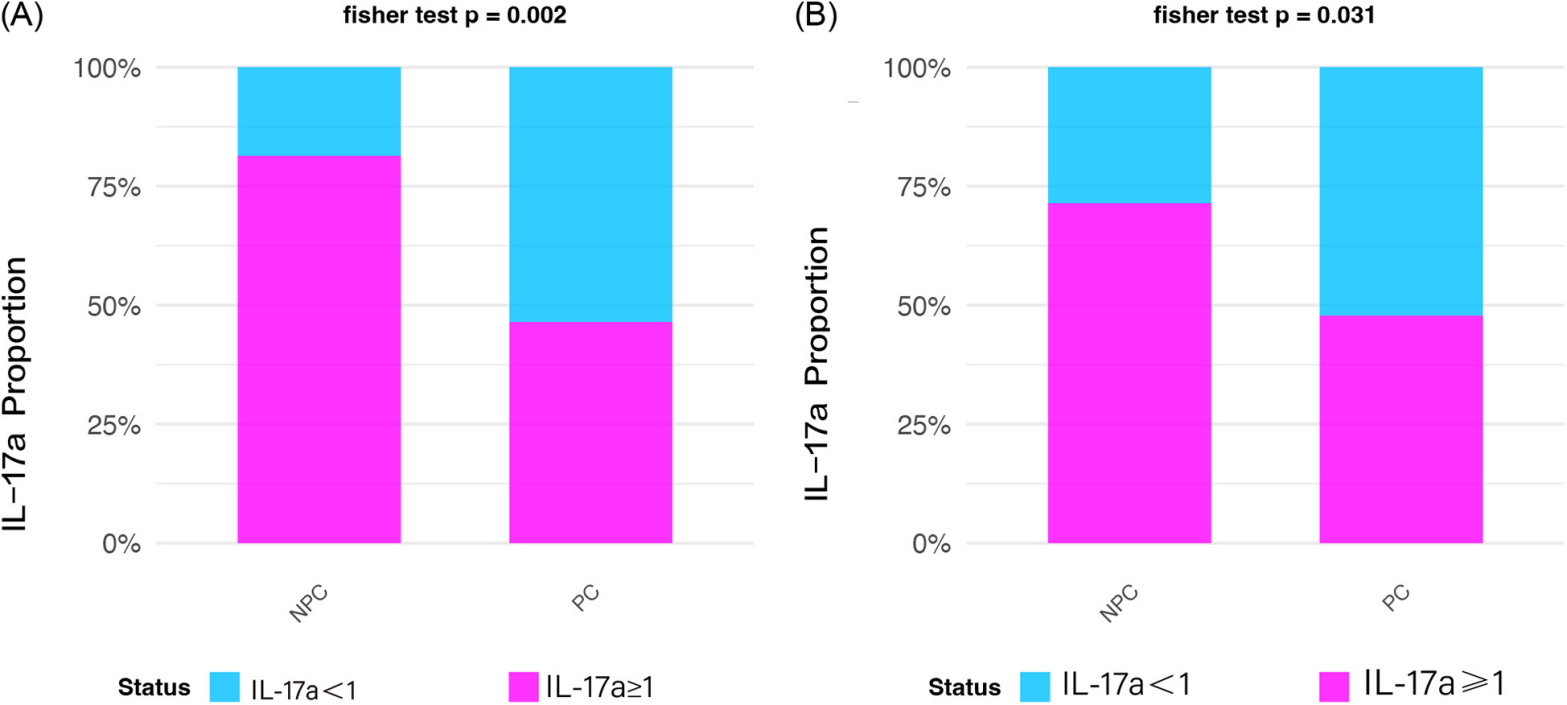
Prevalence of samples with IL-17A ≥1 pg/ml in non-pregnant complications (NPC) and pregnant complications (PC) groups. **(A)** Discovery phase NPC exhibits higher prevalence (*p* = 0.002); **(B)** Validation phase NPC dominance is confirmed (*p* = 0.031).

To ensure analytical robustness, we adopted a dual-method approach for IL-17A assessment. First, continuous IL-17A concentrations were dichotomized using a clinically relevant cutoff (1 pg/ml) and analyzed using Fisher's exact test. In the discovery phase, both statistical methods consistently demonstrated significantly lower IL-17A levels in the PC group compared with controls. The binary analysis revealed that 61.9% (13/21) of PC group samples had IL-17A <1 pg/ml threshold vs. 35.3% (6/17) of the NPC group (Fisher's exact test *p* = 0.002). These findings were replicated in the validation cohort, where 58.3% (14/24) of PC group samples showed IL-17A <1 pg/ml compared with 32.0% (8/25) in the NPC group (Fisher's exact test *p* = 0.031). The intergroup differences were statistically significant (Figure 2).

## 4 Discussion

Immunological dysfunction plays a key role in the pathogenesis of diabetes mellitus, with persistent low-grade inflammation potentially triggering GDM (8, 22). IL-17 exerts its biological effects by specifically binding to the interleukin-17 receptor (IL-17R), thereby promoting inflammation (22). While there is extensive evidence for IL-17's involvement in intrauterine fetal infection, perinatal neonatal infection can trigger fetal IL-17 production, consequently leading to elevated IL-17 levels. In this study, the exploratory phase PC group comprised 11 cases of PI and 10 cases of GDM, while the validation phase PC group included 7 PI cases and 17 GDM cases. Both study phases incorporated key perinatal risk factors associated with elevated IL-17A levels, specifically GDM and PI. Notably, despite employing distinct analytical approaches, the results from both exploratory and validation study phases demonstrated significantly lower umbilical cord blood IL-17A levels in the PC group compared with the NPC group (*p* < 0.05). These findings indicate that, in the context of GDM and perinatal infection, IL-17A levels are not elevated; rather, they were significantly lower in the PC group compared with the NPC group. These findings suggest that neonates born to mothers with GDM and PI may have suppressed cellular immune function at birth. Gestational complications such as GDM and PIs have been shown to significantly alter maternal intestinal homeostasis, including changes in the gut microenvironment and pH levels. These alterations induce gut dysbiosis and disrupt the intestinal microecological balance, subsequently leading to increased circulating endotoxin levels and chronic systemic inflammation. Importantly, these pathological changes may profoundly impact development of the infant gut microbiota and impair establishment of intestinal-specific regulatory T cell (Treg)-mediated immune tolerance. Consequently, pregnant women with these complications exhibit lower IL-17 levels compared with healthy pregnant women (23, 24).

This study demonstrated that umbilical cord blood IL-17A levels were significantly higher in healthy pregnant women than in those with perinatal complications (*p* < 0.05). This finding suggests that maintaining IL-17A below a specific threshold in early life may preserve immune homeostasis at the maternal–fetal interface and support proper immune development in neonates. A longitudinal study (25) of rhesus macaques revealed that TH17 cell populations develop progressively during infancy, with striking interindividual variability in maturation rates. Notably, some infants exhibited severely impaired TH17 development, maintaining negligible frequencies (<1% of CD4+ T cells) through the first year of life—a phenotype mirroring the immunosuppressive states observed in clinical settings. They found that newborn macaques harbor few TH17 cells and that these cells develop progressively throughout the first 18 months of life. Other study demonstrated that the inverse developmental relationship between TH17 and regulatory T cells (Treg) creates an immunosuppressive milieu when IL-17A is low (26). This finding is consistent with the results obtained in our study.

In the context of GDM, diverse immune cell populations—particularly Tregs—undergo spontaneous adaptations to prevent pregnancy disruption (27). Pregnancy represents a unique immunological paradigm characterized by intricate regulatory mechanisms that maintain tolerance toward the semi-allogeneic fetus and support its development. Furthermore, pregnancy creates a window of heightened susceptibility to infections. The placenta, as the first chimeric immune organ to develop, serves as the critical interface where the maternal and fetal circulatory systems are juxtaposed (28). Th17 cells contribute to successful pregnancy outcomes through the secretion of IL-17A and IL-17F, having also been implicated in the pathophysiology of obstetric disorders. IL-17A and IL-17F play pivotal roles in endothelial defense mechanisms, particularly against fungal and bacterial pathogens, and are distributed widely throughout the immune system (29). During gestation, IL-17A is primarily produced by CD4+ T cells in both the peripheral blood and decidual compartments (30). *In vitro* experimental data demonstrate that IL-17A enhances the invasive capacity of JEG-3 trophoblast cells (17, 31). Erika et al. demonstrated that the mean concentration of IL-17A levels was 37.28 pg/ml, peaking during late gestation, and that this peak coincides with a critical developmental window where the fetus (and later the neonate) exhibit immature neuroimmune systems that are both qualitatively and quantitatively distinct from those of adults, resulting in heightened susceptibility to infection. The dynamic balance between Th17 and Treg cells is essential for maintaining immune homeostasis (32): Treg cells expressing IL-10 and TGF-β establish pregnancy tolerance, while Th17 cells producing IL-17, IL-21, and IL-22 are associated with pregnancy loss and autoimmunity. Throughout gestation, effector T cells and their cytokines remain functionally active; however, Treg cells suppress the expression of pro-inflammatory cytokines by Th1 and Th17 cells, thereby improving pregnancy outcomes (18). The findings highlight the dual role of IL-17 in both establishing and maintaining appropriate maternal immune adaptations during pregnancy.

This study reports several innovative findings, including the first observation of a seemingly paradoxical immunological phenomenon in which umbilical cord blood IL-17A levels were significantly lower in the perinatal complications (PC) group compared with the NPC group, suggesting that IL-17A exerts immunoprotective effects below a specific threshold. Methodologically, the study involved discovery and validation phases, combined with dual analytical approaches (nonparametric tests for continuous variables and Fisher's exact test for categorical variables), ensuring robust and reliable results. While our study was primarily exploratory in nature, the observed association between reduced cord blood IL-17A levels and perinatal complications (GDM/PI) may hold important clinical and research implications. These findings suggest IL-17A could potentially serve as a biomarker for identifying neonates at risk of immune dysregulation, which might inform more personalized neonatal care strategies—particularly for high-risk pregnancies where IL-17A screening could help stratify infants needing closer immunological monitoring. While the observational, single-center nature of this study may limit its generalizability, the rigorous selection of a demographically diverse urban population from the Pigman cohort enhances the external validity of our findings. Further multicenter studies are warranted to corroborate these observations. Moving forward, longitudinal studies should examine whether early IL-17A deficiency correlates with subsequent immune-related outcomes in childhood, while mechanistic studies could explore the therapeutic potential of modulating IL-17A pathways in affected neonates.

## 5 Limitations

This study has several inherent limitations that warrant careful consideration. Most notably, the observational study design precludes establishment of causal relationships, as interventional studies manipulating IL-17A levels to observe subsequent risk modification were not conducted. Future larger-scale studies incorporating multivariate regression or propensity score matching—accounting for potential confounders such as birth weight and BMI—are needed to better isolate the effects of perinatal complications on IL-17A levels. The relatively modest sample size (*n* = 87 across both phases) limit the statistical power to detect more subtle associations and could affect the generalizability of our findings. Additionally, as an observational study, our design cannot establish causality between IL-17A levels and perinatal complications, and residual confounding from unmeasured variables (e.g., maternal diet, genetic factors, or environmental exposures) remains a possibility. Considering these constraints, our conclusions should be regarded as preliminary, awaiting validation through larger-scale longitudinal multicenter studies incorporating mechanistic investigations. These limitations notwithstanding, the current findings provide a foundation for future research in this emerging area of investigation.

## 6 Conclusion

The findings from this study demonstrate elevated IL-17 levels in umbilical cord blood from healthy pregnancies compared with those complicated by gestational disorders, suggesting that IL-17A may protect against inflammation during parturition. This implies that, in clinical practice, given the limitations of cord blood sampling prior to delivery, maternal blood monitoring may serve as a surrogate for assessing fetal immune factors, reflecting maternal–fetal immunological status. Analyzing these parameters in postpartum umbilical venous blood could provide a preliminary immunological assessment prior to pathological examination, facilitating effective monitoring of early-life immune system development.

## Data Availability

The original contributions presented in the study are included in the article/Supplementary Material, further inquiries can be directed to the corresponding authors.
